# Detection of *Clostridium difficile* infection clusters, using the temporal scan statistic, in a community hospital in southern Ontario, Canada, 2006–2011

**DOI:** 10.1186/1471-2334-14-254

**Published:** 2014-05-12

**Authors:** Meredith C Faires, David L Pearl, William A Ciccotelli, Olaf Berke, Richard J Reid-Smith, J Scott Weese

**Affiliations:** 1 Department of Population Medicine, University of Guelph, Guelph, Ontario, Canada; 2 Infection Prevention and Control, Grand River Hospital, Kitchener, Ontario, Canada; 3 Department of Pathology and Molecular Medicine, McMaster University, Hamilton, Ontario, Canada; 4 Department of Mathematics and Statistics, University of Guelph, Guelph, Ontario, Canada; 5 Department of Pathobiology, University of Guelph, Guelph, Ontario, Canada

**Keywords:** *Clostridium difficile* infection, Temporal scan statistic, Clusters, Community hospital, Ribotype 027

## Abstract

**Background:**

In hospitals, *Clostridium difficile* infection (CDI) surveillance relies on unvalidated guidelines or threshold criteria to identify outbreaks. This can result in false-positive and -negative cluster alarms. The application of statistical methods to identify and understand CDI clusters may be a useful alternative or complement to standard surveillance techniques. The objectives of this study were to investigate the utility of the temporal scan statistic for detecting CDI clusters and determine if there are significant differences in the rate of CDI cases by month, season, and year in a community hospital.

**Methods:**

Bacteriology reports of patients identified with a CDI from August 2006 to February 2011 were collected. For patients detected with CDI from March 2010 to February 2011, stool specimens were obtained. *Clostridium difficile* isolates were characterized by ribotyping and investigated for the presence of toxin genes by PCR. CDI clusters were investigated using a retrospective temporal scan test statistic. Statistically significant clusters were compared to known CDI outbreaks within the hospital. A negative binomial regression model was used to identify associations between year, season, month and the rate of CDI cases.

**Results:**

Overall, 86 CDI cases were identified. Eighteen specimens were analyzed and nine ribotypes were classified with ribotype 027 (n = 6) the most prevalent. The temporal scan statistic identified significant CDI clusters at the hospital (n = 5), service (n = 6), and ward (n = 4) levels (P ≤ 0.05). Three clusters were concordant with the one *C. difficile* outbreak identified by hospital personnel. Two clusters were identified as potential outbreaks. The negative binomial model indicated years 2007–2010 (P ≤ 0.05) had decreased CDI rates compared to 2006 and spring had an increased CDI rate compared to the fall (P = 0.023).

**Conclusions:**

Application of the temporal scan statistic identified several clusters, including potential outbreaks not detected by hospital personnel. The identification of time periods with decreased or increased CDI rates may have been a result of specific hospital events. Understanding the clustering of CDIs can aid in the interpretation of surveillance data and lead to the development of better early detection systems.

## Background

*Clostridium difficile* represents a significant burden to public health in terms of outbreaks, infection control measures, increased patient morbidity and mortality rates, and patient costs [1]. Detection of *C. difficile* outbreaks in healthcare settings may rely on rule-based or threshold criteria; criteria that are prone to error as they fail to address changes in population size or random variation [2]. The identification of a spurious disease cluster may result in the waste of hospital resources due to investigational procedures and/or interventions [2]. Conversely, a delay in recognizing, or a failure to recognize, a true disease cluster can inhibit the application of enhanced infection control measures early in an outbreak, with the potential to prolong the outbreak. Understanding the clustering of infectious diseases, spatially and/or temporally, can be used to identify risk factors [3], facilitate detailed investigations to determine the association between exposures and disease interventions [4], and detect outbreaks [5]. A commonly used statistical technique to detect disease clusters, the scan statistic has been employed to investigate a wide array of infectious diseases or pathogens including *Shigella*[6], malaria [7], meningococcal disease [8], *Escherichia coli* 0157 [5], and listeriosis [9].

There has been limited incorporation of the scan statistic for the detection and evaluation of spatial and/or temporal disease clusters for hospital surveillance purposes. Furthermore, no studies have been conducted in community hospitals evaluating the effectiveness of the temporal scan statistic, compared to traditional surveillance techniques, for the detection and evaluation of *C. difficile* clusters. Understanding the clustering of *C. difficile* infections (CDI) can aid in the interpretation of ongoing surveillance data, detect outbreaks at an earlier time, and lead to new hypotheses regarding the transmission of *C. difficile* within the hospital setting.

The objectives of this study were the following: to investigate the utility of the temporal scan statistic for detecting CDI clusters in a community hospital and validate statistically significant clusters using molecular typing (i.e., ribotyping) and hospital records concerning known CDI outbreaks; and to determine if there were significant differences in the rate of CDI cases by month, season, and year using regression models.

## Methods

### Study site

A single community hospital in southern Ontario, Canada participated in this study. This facility has 345 beds and over 200,000 in- and out-patient visits annually and provides a number of services including oncology, pediatrics, intensive care, emergency, internal medicine, psychiatry, rehabilitation, and surgery. This study was approved by the research ethics boards of the University of Guelph and the participating hospital.

### Case definition

For this investigation, a case of CDI was defined as a patient with new diarrhea and any of the following (WAC, personal communication):

• Laboratory confirmation of a positive fecal toxin assay for *C. difficile*; and/or

• Visualization of pseudomembranes on sigmoidoscopy or colonoscopy; and/or

• Histological/pathological diagnosis of pseudomembranous colitis.

Diarrhea was characterized as the following: three or more bowel movements in 24 hours; loose/watery bowel movements that conform to the shape of the specimen container; and there is no other recognized etiology for the diarrhea. Cases of CDI were classified as healthcare-associated if the patient’s diarrhea developed > 48 hours following hospital admission.

### Data collection

For this longitudinal study, a case was included if *C. difficile* was identified between August 1, 2006 and February 28, 2011 and > 48 hours following hospital admission. Only one CDI case per patient per admission-discharge period was included in the analyses. The admission-discharge period was defined as the interval from when a patient was admitted to, and discharged from, the hospital. Transfer to another ward was not considered a discharge. For a patient to be admitted ≥ 2 times to the hospital, > 24 hours between the discharge and admission dates was required. Data from the first bacteriology report per patient per admission-discharge period were obtained. Bacteriology reports from CDI patients located in the emergency and hemodialysis departments were excluded as these departments specifically support outpatients.

For CDI cases identified between March 1, 2010 and February 28, 2011, the hospital’s microbiology laboratory collected and submitted stool specimens that were positive for *C. difficile* for molecular typing. In the participating hospital, *C. difficile* was identified by testing for the *C. difficile* antigen, glutamate dehydrogenase, and toxins A and B (C. Diff Quik Chek Complete, TechLab, Blacksburg, Virginia, USA). If a specimen was positive for the *C. difficile* antigen, but toxin negative, a second test was conducted to detect toxins A and B (Immuno*Card* Toxins A and B, Meridian Bioscience, Inc., Cincinnati, Ohio, USA). This particular testing regimen was adopted by the hospital’s microbiology laboratory, as an additional 5-10% of specimens were identified as *C. difficile* by incorporating the second test (WAC, personal communication). At the hospital level, all stool specimens submitted for *C. difficile* testing were collected at the discretion of medical personnel. Only one specimen per patient was collected for molecular typing.

Information collected from the bacteriology report included a unique patient identifier, dates pertaining to when the patient was admitted and discharged, when a stool specimen was collected for *C. difficile* testing, and the ward location of the patient when the stool specimen was collected. For ward location, bacteriology reports provided both service and ward designations. Services were defined as specific departments (e.g., internal medicine, surgery) whereas wards were characterized as specific, physically distinct units that comprised a service (e.g., S1 and S2 wards comprised the surgery department).

Information regarding the number of patient days per month for each service was collected. For wards, data on patient days were obtained only from those wards that were operational and provided the same service for the entire study period (i.e., 55 months). For descriptive statistics, incidence rates for CDI were expressed as the number of CDI cases per 10,000 patient days.

Data pertaining to known *C. difficile* outbreaks that occurred during the study period (e.g., start and end date, ward location, and number of patients involved) were collected from the hospital’s Infection Prevention and Control (IPC) department. Culture and molecular typing were not performed as a clinical or infection control tool, therefore historical typing data were not available. In the participating hospital, outbreak notification thresholds for *C. difficile* are employed and consist of the following [10]:

• For units with ≥ 20 beds: 3 cases of CDI identified on one unit within a 7 day period or 5 cases within a 4 week period. For units with < 20 beds: 2 cases of CDI identified on one unit within a 7 day period or 4 cases within a 4 week period; or

• A baseline CDI rate for 2 months that is at or above the 80^th^ percentile for comparator hospitals; or

• A facility rate that is ≥ 2 standard deviations above their baseline.

### Processing of specimens

Patient stool specimens were obtained from the hospital’s microbiology laboratory following *C. difficile* confirmation and forwarded to the laboratory at the University of Guelph on a weekly basis. Approximately 1 gm of feces was inoculated into 9 ml of brain-heart infusion broth supplemented with 0.1% sodium taurocholate and incubated, anaerobically, at 37°C for 5 days. A 2 ml aliquot of broth was alcohol shocked by addition of an equal volume of anhydrous alcohol and incubated at room temperature for one hour followed by centrifugation at 4,000 rpm for 10 minutes. The resulting pellet was inoculated onto *C. difficile* moxalactam-norfloxacin agar (Oxoid, Nepean, Ontario, Canada) and incubated anaerobically for 24–96 hours at 37°C. Presumptive colonies were sub-cultured onto blood agar (Oxoid, Nepean, Ontario, Canada) and identified as *C. difficile* based on characteristic morphology, odour, and production of L-proline-aminopeptidase (Prodisk, Remel, Lenexa, Kansas, USA). All *C. difficile* isolates were investigated for the presence of genes for toxin A (*tcdA*) [11], toxin B (*tcdB*) [12], and the binary toxin (*cdtA*) [13] using PCR. Ribotyping [14] was also performed on all *C. difficile* isolates. When a ribotype pattern was known to be an international ribotype based on comparison to reference strains, the appropriate numerical designation (e.g., 027) was assigned. Otherwise, an internal laboratory designation was assigned.

### Statistical analysis

All bacteriology reports were provided by the hospital in electronic format. The temporal scan statistic was performed using SaTScan version 9.0 [15] and all descriptive statistics and model building were conducted using Stata 11.0 (StataCorp LP, College Station, Texas, USA). For all hypothesis tests, if not stated otherwise, a 5% significance level was applied (α ≤ 0.05).

### Temporal scan statistic to identify CDI clusters

To identify CDI clusters, the temporal scan statistic employing a Poisson model was used. Currently, there are no versions of the scan statistic that use the negative binomial distribution for its likelihood ratio tests. However, it should be noted that Poisson models are a special case of negative binomial models which have an additional overdispersion parameter. In the case of the scan statistic, the P-values of the test are based on Monte-Carlo hypothesis testing rather than distributional assumptions. Consequently, the P-values of the scan statistic are estimated correctly without the addition of an overdispersion parameter.

The scan statistic involves a flexible scanning window that gradually moves across time. The number of observed and expected observations inside the window is compared to outside the window, at each time period, with the greatest excess of observed cases noted [15,16]. Under the null hypothesis, the expected number of cases in each time period covered by the scanning window is proportional to its population size; whereas under the alternative hypothesis, there is an elevated risk within the window as compared to outside the window [15].The window identified as least likely due to chance is subsequently evaluated by a maximum likelihood test with a test decision based on a Monte-Carlo simulated P-value [15]. For this analysis, Monte-Carlo replications were set at 9999.

To detect CDI clusters, only periods with high rates (i.e., a one-tailed test) were scanned. The maximum temporal window size was set to 50% of the study period. In addition, an adjustment for more likely clusters was made by conducting an iterative scan statistic where the cluster identified from the first iteration is removed from the data set and a new analysis is performed using the remaining data [15]. Data were analyzed on a monthly scale. A cluster was defined as a period where the rate of CDI cases was statistically higher than expected inside a window compared to outside a window.

Retrospective monthly scan tests were conducted for the entire study period (i.e., August, 2006 to February, 2011) as well as annually (January 1^st^ – December 31^st^) from 2006 to 2011. For 2006, the time interval was restricted from August 1^st^ – December 31^st^ and for 2011, the time interval was confined to January 1^st^ – February 28^th^. For each time interval, temporal scan tests were conducted to identify CDI clusters at three different levels including hospital wide, service, and ward. For this analysis, 10 services were identified and included acute care, complex care, hospice, the intensive care unit, internal medicine, oncology, pediatrics, psychiatry, rehabilitation, and surgery. Three wards were identified and included M1 (medicine), S1 (surgery), and S2 (surgery).

Significant (P ≤ 0.05) CDI clusters that were identified by the temporal scan statistic were compared to outbreaks identified by the IPC department. In addition, CDI cases that comprised significant *C. difficile* clusters were compared based on ribotype. Characteristics of significant clusters (e.g., time frame, observed and expected case numbers, P-value, and ribotype) are reported.

### Statistical model for CDI rates

To evaluate the association between the rate of CDI cases in the hospital and the independent variables year, season, and month, a Poisson regression analysis was conducted. For the exposure variable year, 2011 was removed from the analysis as no CDI cases were identified during that period. For season, months were categorized in the following groupings: winter (January – March), spring (April – June), summer (July – September), and fall (October – December). The dependent variable was the number of CDI cases and the offset was the natural log of the population at risk (i.e., number of patient days), for a particular month. Due to the hierarchical structure of the data, CDI cases nested in wards, a multilevel Poisson model including a random intercept for ward and a fixed effect for service, was used to adjust for clustering. The variable service was categorized as medicine (intensive care, oncology, pediatrics, internal medicine), surgery, and other (psychiatry, rehabilitation, hospice, childbirth, nursery).

The Spearman’s rank correlation coefficient was used to identify correlations between independent variables. Variables with a correlation above 0.8 were investigated for collinearity and the biologically more plausible variable was retained in the model [17]. Univariable multilevel Poisson models were fit using marginal likelihood estimation via the adaptive quadrature algorithm (as implemented in the xtmepoisson procedure in Stata) to screen each independent variable with the dependent variable using a liberal significance level (α ≤ 0.20). Manual backwards step-wise modeling was applied to fit a multivariable multilevel Poisson model to all previously identified significant co-variables. To assess the significance of the independent variables, the likelihood ratio test was utilized. Confounding was evaluated by examining the effect of the removed variables on the coefficients of the remaining variables. A variable was considered to be a confounder if it changed the model coefficients by ≥ 20% [18]. Interaction terms were examined for all independent variables, with statistically significant main effects retained in the model. Due to concerns regarding unexplained overdispersion, the Poisson random effects model was compared to a negative binomial random effects model using Akaike’s Information Criteria (AIC). The random effects negative binomial model was used where the overdispersion parameter was allowed to vary randomly by cluster based on a beta distribution (using the xtnbreg command in Stata) [19]. The model with the lowest AIC was selected. Based on the final multivariable model, contrasts for independent variables with >2 categories were examined to investigate significant differences between any two categories.

## Results

### Descriptive statistics

Over the study period, 86 CDI cases were identified, contributing 5,499 patient-days. No patient was identified with CDI for two or more hospital admission-discharge periods. Forty-six (53.5%) cases were male and 40 (46.5%) cases were female. For males, cases ranged in age from 21 to 95 years (median = 72 years) and for females, cases ranged in age from 25 to 100 years (median = 76 years).

**Figure 1:**
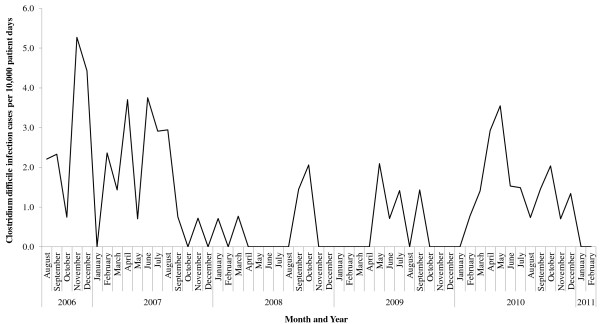
**Incidence rate of *Clostridium difficile *** **infection cases from August 1, 2006 to February 28, 2011.**

The monthly incidence rate of CDIs fluctuated over the study period and ranged from 0 to 5.28 CDI cases/10,000 patient days with an average of 1.14 CDI cases/10,000 patient days (Figure 1). Based on the surveillance data that were available, no CDI cases were identified in the first two months of 2011. Summary characteristics of the CDI incidence rate according to month, year, season, service, and ward are presented in Table 1. The highest incidence rate for CDI occurred in 2006, followed by 2007 and 2010. April and May had the highest incidence rates, 1.66 and 1.59 CDI cases/10,000 patient days, respectively. For season, spring was observed with the highest incidence rate for CDI and winter was observed with the lowest. Services with the largest CDI incidence rates included acute care, the intensive care unit, and internal medicine. No CDI cases were reported in the hospice or pediatric wards.

**Table 1:** Summary characteristics of 86 CDI cases, August 1, 2006 to February 28, 2011

**CDI characteristics**	**Incidence rate of CDI (cases per 10,000 patient days)**
Month^1^	
January	0.18
February	0.79
March	0.90
April	1.66
May	1.59
June	1.50
July	1.45
August	1.18
September	1.49
October	0.70
November	1.50
December	1.11
Year^2^	
2006 (August – December)	2.99
2007 (January – December)	1.58
2008 (January – December)	0.42
2009 (January – December)	0.47
2010 (January – December)	1.49
2011 (January – February)	0
Season^1^	
Spring (April – June)	1.6
Summer (July – September)	1.4
Fall (October – December)	1.1
Winter (January – March)	0.6
Service^3^	
Acute care	3.4
Complex care	0.5
Hospice	0
Intensive care unit	2.7
Internal medicine	2.2
Oncology	0.8
Pediatrics	0
Psychiatry	1.0
Rehabilitation	0.9
Surgery	1.6
Ward^3^	
M1	3.4
S1	1.7
S2	1.5

CDI = *Clostridium difficile* infection.

^1^Incidence rate presented is based on an average for that specific period.

^2^Incidence rate presented is the total for that specific year.

^3^Incidence rate presented is the total for that service or ward from August 1, 2006 to February 28, 2011.

From March 1, 2010 to February 28, 2011, 24 CDI cases were identified and 18 (75%) *C. difficile* specimens were collected for ribotyping (Table 2). Overall, nine different ribotypes were identified with ribotype 027 (33.3%) being the most prevalent. Nine (50%) of the *C. difficile* isolates were identified with the gene for the binary toxin.

**Table 2:** **Typing data for 18 *Clostridium difficile *** **patient isolates, March 1, 2010 to February 28, 2011**

**Ribotype**	**Number of *Clostridium difficile *** **isolates (%)**	**Toxin genes**
027	6 (33.3)	*tcdA, tcdB, cdtA*
001	3 (16.7)	*tcdA, tcdB*
V	2 (11.1)	*tcdA, tcdB*
Y	2 (11.1)	*tcdA, tcdB, cdtA*
AF	1 (5.6)	*tcdA, tcdB*
T	1 (5.6)	*tcdA, tcdB*
CMFA	1 (5.6)	*tcdA, tcdB*
CMFB	1 (5.6)	*tcdA, tcdB*
CMFC	1 (5.6)	*tcdA, tcdB, cdtA*

*tcdA* = Toxin A.

*tcdB* = Toxin B.

*cdtA* = Binary toxin.

### Temporal scan statistic to identify CDI clusters

Over the study period, the temporal scan statistic identified significant CDI clusters at the hospital (n = 5), service (n = 6), and ward (n = 4) levels (Table 3). As separate scan tests were conducted at various levels, it was observed that several clusters overlapped in time and/or location. Of the 15 clusters identified, only five were classified as separate events.

**Table 3:** Statistically significant temporal clusters of CDI rates, August 1, 2006 to February 28, 2011

**Cluster number**	**Period scanned**^**1**^	**Date of cluster (year/month/day)**	**Number of cases**	**Observed/Expected**	**P-value**	**Ribotype (n)**
**Hospital wide scans**						
1^a^	2006-2011^3^	2006/8/1 – 2007/8/31	44	2.22	< 0.001	Not available
2^b^	2006-2011^3^	2010/3/1 – 2010/12/31	24	2.39	0.001	027 (6), 001 (3), MOH-V (2),
						MOH-Y (2),
						MOH-AF (1),
						MOH-T (1),
						CMFA (1),
						CMFB (1),
						CMFC (1)
3^c^	2007^4^	2007/4/1 – 2007/8/31	19	1.75	0.025	Not available
4^d^	2008^5^	2008/9/1 – 2008/10/31	5	4.22	0.041	Not available
5	2009^4^	2009/5/1 – 2009/9/30	8	2.39	0.006	Not available
**Service scans**						
6^b^	Complex care,	2010/4/1 – 2010/5/31	4	10.32	0.04	CMFA (1), CMFB (1), MOH-V (1), 001 (1)
	2006-2011^4,6^					
7^c^	Intensive care unit,	2007/6/1 – 2007/7/31	4	12.14	0.019	Not available
	2006-2011^4, 7^					
8^a^	Internal medicine,	2006/8/1 – 2007/8/31	18	2.82	< 0.001	Not available
	2006-2011^3^					
9^c^	Internal medicine, 2007^4^	2007/4/1 – 2007/8/31	10	2.37	0.001	Not available
10^d^	Internal medicine, 2008^5^	2008/9/1 – 2008/10/31	3	5.78	0.026	Not available
11^a^	Psychiatry,	2006/8/1 – 2007/9/30	9	4.23	< 0.001	Not available
	2006-2011^3^					
**Ward scans**						
12^a^	M1, 2006-2011^3^	2006/8/1 – 2007/8/31	14	3.41	< 0.001	Not available
13^d^	M1, 2006-2011^5,8^	2008/9/1 – 2008/10/31	3	15.17	0.017	Not available
14^c^	M1, 2007^4^	2007/4/1 – 2007/8/31	9	2.37	0.004	Not available
15^a^	S1, 2006-2011^4^	2006/11/1 – 2006/12/31	4	9.83	0.042	Not available

CDI = *Clostridium difficile* infection.

n = Number of isolates.

^1^Time period over which the scan was conducted.

^2^Month and year the significant cluster was identified by the temporal scan statistic.

^3^Long duration cluster (7–14 months in length).

^4^Short duration cluster (1–6 months in length).

^5^Cluster was part of the *C. difficile* outbreak identified by Infection Prevention and Control personnel.

^6^Cluster was also identified in the Complex care department for the 2010 annual analysis.

^7^Cluster was also identified in the Intensive care unit for the 2007 annual analysis.

^8^Cluster was also identified in M1 in the 2008 annual analysis.

^a-d^Indicates a cluster identified by >1 temporal scan.

Overall, clusters ranged in length from 2 to 14 months in duration (median = 5 months) and involved a range of 3 to 44 CDI cases (median = 9 cases) per cluster. From August 2006 to February 2011, IPC personnel identified only one *C. difficile* outbreak which occurred during a three-and-a-half week period during October and November of 2008. This outbreak was identified by three scan tests applied at the hospital, service, and ward levels (Cluster ID 4, 10, 13). However, for all three clusters, the starting and end dates spanned from September to October 2008, respectively.

For the remaining CDI clusters, overall, 7 (58.3%) were considered to be of short duration (1–6 months in length) and 5 (41.7%) were classified as long duration (7–14 months in length). Investigation of the short duration cluster in ward M1 (Cluster ID 14) revealed that three of the CDI cases were identified over a two day period and therefore met one of the threshold criteria for a possible *C. difficile* outbreak in this facility. As this cluster was identified using historical data, molecular data were not available for validation purposes.

Two clusters (Cluster ID 2, 6) were characterized using typing data. For both clusters, analysis of *C. difficile* specimens identified several different ribotypes present in the patient population. For the long duration, hospital wide cluster (Cluster ID 2), further examination of the six ribotype 027 cases revealed that three of the cases were located in the same ward and were identified with CDI over a 20 day period. Furthermore, two of the ribotype 027 cases were identified on the same day, therefore meeting one of the hospital’s threshold criteria for a possible *C. difficile* outbreak. For the remaining cluster with corresponding typing data (Cluster ID 6), all four cases were identified with dissimilar ribotypes, and these cases were not concordant with any of the hospital’s notification criteria. For all other clusters identified by the temporal scan statistic, none met the notification threshold criteria used by the study hospital.

### Statistical model for CDI rates

A random effects negative binomial model was chosen over the random effects Poisson model based on the AIC value. Results of the univariable multilevel negative binomial regression models indicated that year, season, and month were significantly associated with the rate of CDI cases (Table 4). For the final multivariable multilevel negative binomial model, year and season were significant independent variables (Table 5). The final model indicated that the years 2007–2010 were significantly associated with a decreased incidence rate of CDI compared to 2006 and that the rate of CDI cases was significantly higher in the spring compared to the fall. Results from significant model-based contrasts indicated an increase in the incidence rate in CDI cases in the spring compared to the winter, and in the years 2007 and 2010 compared to 2008 and 2009 (Table 6).

**Table 4:** Univariable regression analysis* of variables associated with the rate of CDI cases

**Variable**	**Description**	**IRR**	**95% CI**	**P-value**
Year	2006	Referent		
	2007	0.48	0.26 – 0.89	0.019
	2008	0.13	0.05 – 0.32	< 0.001
	2009	0.16	0.07 – 0.36	< 0.001
	2010	0.49	0.27 – 0.90	0.022
Season	Fall	Referent		
	Winter	0.54	0.25 – 1.18	0.121
	Spring	1.33	0.73 – 2.42	0.356
	Summer	1.15	0.63 – 2.07	0.652
Month	January	Referent		
	February	4.19	0.47 – 37.55	0.200
	March	4.76	0.54 – 41.95	0.160
	April	7.52	0.89 – 62.97	0.063
	May	8.70	1.09 – 69.11	0.041
	June	7.55	0.92 – 61.69	0.059
	July	6.36	0.75 – 53.59	0.089
	August	6.19	0.77 – 50.00	0.087
	September	7.95	1.01 – 62.47	0.049
	October	5.01	0.59 – 41.95	0.137
	November	6.90	0.86 – 55.33	0.069
	December	6.11	0.76 – 49.29	0.089
Service	Medicine^1^	Referent		
	Surgery	1.29	0.27 – 6.31	0.747
	Other^2^	1.02	0.29 – 3.61	0.973

*Multilevel negative binomial regression model with a random intercept for ward.

CDI = *Clostridium difficile* infection.

IRR = Incidence rate ratio.

CI = Confidence interval.

^1^Included the following departments: intensive care (adult and neonatal), oncology, pediatrics, and internal medicine.

^2^Included the following departments: psychiatry, rehabilitation, hospice, childbirth, and nursery.

**Table 5:** Multivariable regression model* of variables associated with the rate of CDI cases

**Variable**	**Description**	**IRR**	**95% CI**	**P-value**
Year	2006	Referent		
	2007	0.43	0.22 – 0.83	0.013
	2008	0.11	0.04 – 0.29	< 0.001
	2009	0.14	0.06 – 0.32	< 0.001
	2010	0.43	0.22 – 0.83	0.012
Season	Fall	Referent		
	Winter	0.81	0.36 – 1.80	0.606
	Spring	2.07	1.10 – 3.89	0.023
	Summer	1.29	0.73 – 2.29	0.379
Service	Medicine^1^	Referent		
	Surgery	1.15	0.20 – 6.53	0.871
	Other^2^	0.74	0.18 – 2.95	0.667

^*^Multivariable multilevel negative binomial regression model with a random intercept for ward.

CDI = *Clostridium difficile* infection.

IRR = Incidence rate ratio.

CI = Confidence interval.

^1^Included the following departments: intensive care (adult and neonatal), oncology, pediatrics, and internal medicine.

^2^Included the following departments: psychiatry, rehabilitation, hospice, childbirth, and nursery.

**Table 6:** Significant model-based contrasts between the rate of CDI cases and year and season

**Description**	**IRR**	**95% CI**	**P-value**
2007 versus 2008	3.82	1.60 – 9.12	0.002
2007 versus 2009	3.15	1.39 – 7.08	0.006
2010 versus 2008	3.85	1.61 – 9.21	0.002
2010 versus 2009	3.17	1.42 – 7.08	0.005
Spring versus Winter	2.56	1.22 – 5.38	0.013

CDI = *Clostridium difficile* infection.

IRR = Incidence rate ratio.

CI = Confidence interval.

## Discussion

In public health, the detection of outbreaks generally depends on non-statistical methods, *ad hoc* analyses, or unvalidated thresholds [6]. Furthermore, within the hospital setting, rule-based criteria are often applied to ascertain if an outbreak has occurred [2]. Consequently, the above approaches may result in false outbreak alarms or outbreaks that are overlooked, and subsequently the misuse of hospital resources or a missed opportunity for further case investigation [2] or prompt intervention, prevention, and control. Therefore, studies evaluating the incorporation of various statistical methods to complement traditional surveillance techniques within the hospital setting are being performed. For *C. difficile*, research has been conducted to assess the effectiveness of different statistical methods, including the Knox test [20,21] and computer-assisted algorithms using microbiology data [22] to enhance surveillance in healthcare facilities. The Knox test focuses on clustering in time and space [20] and does not utilize a scanning window. For the two studies incorporating this statistical technique, results were not compared to routine infection control strategies for identifying *C. difficile* infections within the hospital setting [20,21]. Furthermore, the lack of patient specimens in one investigation precluded the validation of *C. difficile* clusters at the molecular level [21]. Although Rexach and colleagues [20] conducted fingerprint analysis on *C. difficile* isolates from a pediatric patient population, the researchers observed that none of the clusters based on molecular typing corresponded to clusters identified by the Knox test. Through the creation of a monitoring system using microbiological data, Hacek and colleagues [22] identified several suspected outbreaks, of various pathogens, in a tertiary-care hospital that were not detected by standard surveillance techniques. However, a large majority of the suspected outbreaks were not investigated using molecular typing.

The application of the scan statistic to detect *C. difficile* clusters has not been evaluated. The present study is among the first investigations to assess the utility of the temporal scan statistic for detecting *C. difficile* clusters in a community hospital in addition to investigating clusters using molecular techniques.

### CDI clusters

In this investigation, a cluster was defined as a statistically significant high rate of CDI cases within a time period. The application of the temporal scan statistic to historical hospital data resulted in the identification of 15 significant clusters, five of which were separate events. By conducting scan tests at the hospital, service, and ward levels, this methodology allowed for the identification of clusters in different departments and wards. Subsequently, investigations can be focused at various levels to identify specific factors that may be associated with an increase in the rate of CDI cases in addition to developing and evaluating intervention and/or prevention measures.

Data from the hospital’s IPC department indicated that during the study period, only one *C. difficile* outbreak was identified by hospital personnel. Although this particular outbreak was identified at three different levels (Cluster ID 4, 10, 13) using the temporal scan statistic, the starting date for the clusters was reported as September 2008, a month earlier than the outbreak noted by IPC personnel. This is an important finding as the outbreak may have begun in September and therefore, case investigations and institution of infection control measures to prevent transmission events and environmental contamination with *C. difficile*, could have been initiated earlier.

Ten clusters were identified between August 2006 and February 2010, the retrospective study period; therefore, molecular data were not available to validate these events. Further examination of these 10 events revealed four clusters that were considered to be of long duration and six clusters considered to be of short duration. For the long duration clusters (Cluster ID 1, 8, 11, 12), a significant increase in the rate of CDI cases were noted in the hospital overall, which was attributed to increases in the rate of CDI cases in two services (e.g., internal medicine and psychiatry) and one ward that was located in the internal medicine department. Although the exact biological relevance of these long duration clusters is not known, plausible scenarios include extended outbreaks, temporal trends, and the representation of systematic changes at the hospital level during the surveillance period. Results from the statistical analyses indicated that a significant increase in the CDI incidence rate occurred during 2007 as compared to 2008 and 2009, which was concordant with the temporal scan statistic as all four long duration clusters spanned 2007. The application of standardized reporting procedures for CDI in the province of Ontario is a possible explanation for this finding. Commencing September 2008, hospitals located in Ontario were required to collect and report monthly data on CDI to the Ontario Ministry of Health and Long-term Care for posting on a public accessible web site [23]. Daneman and colleagues [24] assessed the change in hospital-specific rates of CDI prior to and following the mandatory reporting period. Before September 2008, the overall rates of CDI in Ontario increased from 7.01 cases/10,000 patient days in 2002 to 10.79 cases/10,000 patient days in 2007. Following September 2008, there was a 26.7% reduction in CDI rates over the reporting period. This decrease in the CDI rate may be attributable to hospitals strictly adhering to best practices in *C. difficile* prevention due to the mandatory reporting of rates to the public [24].

For short duration clusters (Cluster ID 3, 5, 7, 9, 14, 15), a significant hospital wide cluster in 2007 (Cluster ID 3) was a result of an increase in the rate of CDI cases in two services (e.g., the intensive care unit and internal medicine) and a ward located in the internal medicine department. Further examination of this particular cluster revealed a potential *C. difficile* outbreak in this ward that was not identified by IPC personnel. In addition, a review of three other CDI cases that comprised this ward cluster revealed that these three patients were identified with CDI over a 20 day period. Although investigations of the remaining short duration clusters did not reveal additional potential outbreaks consistent with the hospital’s threshold criteria, overall, these clusters should not be discounted as potential outbreaks on the basis of not conforming to specific criteria. In the study hospital, the threshold criteria used for detecting a CDI outbreak are provided by the province’s Ministry of Health and Long-Term Care [10]; however, as these thresholds have not been validated, the criteria are subjective guidelines.

In this investigation, two clusters were investigated using molecular data. For the cluster observed in the complex care service (Cluster ID 6), four CDI cases were identified over a two month period, with three of the cases specifically identified within a 16 day period. Molecular typing of the *C. difficile* isolates established that each case had a different ribotype. Based on these results, it is difficult to determine the biological relevance of this particular cluster. Simply, this event may represent a coincidental occurrence of CDI cases with different strains; however, this cluster may also represent a potential outbreak or possible transmission events. It has been previously reported that different ribotypes may be present in a patient population during a *C. difficile* outbreak [25]. Additionally, transmission of *C. difficile* due to environmental contamination or by unidentified patients, staff, or visitors infected or colonized with *C. difficile* are also possible scenarios. In one study investigating CDIs in healthcare and community settings, 45% (428/957) of CDI cases were genetically distinct from previous CDI cases indicating that *C. difficile* was not transmitted from another symptomatic patient but may have been acquired from asymptomatic individuals or other reservoirs [26]. The second cluster that was investigated with molecular data (Cluster ID 2) contained 24 CDI cases which were identified hospital wide over a 10 month period. Molecular typing of *C. difficile* specimens from 18 cases identified nine different ribotypes in this cluster, including ribotype 027. Further examination of three of the ribotype 027 cases identified a potential outbreak in a ward, based on the hospital’s threshold criteria.

### CDI rate

Results of the statistical analyses indicate that the incidence rate for CDI was significantly higher in the spring compared to the fall and winter seasons. The identification of increased CDI rates in spring is in contrast to previous studies that demonstrated an increase in the incidence rate of CDI cases in the winter months [27-29]. However, in these investigations, data pertaining to season (e.g., what months reflected a particular season) were not defined or analyses of specific individual seasons (e.g., winter, spring, summer, fall) were not performed. The higher incidence rates of CDI in the winter months may be attributed to various determinants including the presence of the influenza virus or respiratory syncytial virus in the hospital patient population [27], the use of antimicrobials [30-32] especially in the winter months due to respiratory viruses [29] and co-morbidities or severe illness in patients admitted to the hospital during winter months [29].

Model-based contrasts for the independent variable year identified 2010 as being significantly associated with an increase CDI incidence rate compared to years 2008 and 2009. This increased CDI rate in the hospital in 2010 may be attributable to the 2009–2010 H1N1 influenza pandemic. As H1N1 cases were admitted to the participating hospital, it is possible that infections due to *C. difficile* increased soon after as routine infection control strategies focused on H1N1 instead of *C. difficile*.

In this investigation, nine different ribotypes were identified in the patient population, with ribotypes 027 and 001 being the most prevalent. This is not surprising as these two ribotypes have been identified as the most prevalent among *C. difficile* isolates from Ontario diagnostic laboratories [33]. Ribotype 027 has been responsible for various outbreaks of CDI with increased severity, high relapse rates, and significant mortality in Canada [34,35] and internationally [36,37]. Overall, 50% of *C. difficile* isolates in this study were identified containing the gene for the binary toxin. The role of the binary toxin in the pathogenesis of *C. difficile* is unclear.

In the present investigation, retrospective analyses of microbiology data using a statistical technique were promising in terms of identifying plausible *C. difficile* clusters. However, there is a need for prospective studies to identify statistical *C. difficile* clusters, assess the incidence of false positive clusters, investigate the detection of outbreaks at an earlier time, and evaluate how much this form of quantitative surveillance supports and improves traditional hospital surveillance methodologies.

This study has several limitations. Patient isolates were only collected over one year and not all patient specimens were available for typing. Therefore, the true molecular composition of clusters, and the biological relevance of several clusters, was unknown. Some of the clusters/temporal patterns identified during this investigation could represent shifts in patient demographics. However, patient demographic data (i.e., sex, age) of the hospital population were not available at the temporal resolution required for our analyses. Furthermore, the number of *C. difficile* cases included in the analysis was limited and the investigation was conducted in only one community hospital. Consequently, results may not be generalizable to other medical facilities.

## Conclusions

Epidemiological data containing a time reference can be used to perform temporal analyses for the detection and evaluation of CDI clusters. By understanding the clustering of CDIs in time, potential risk factors and/or outbreaks may be identified. Furthermore, by incorporating molecular typing data with epidemiological data, CDI clusters can be further examined to better identify potential outbreaks, prevent the misclassification of cases as outbreak or non-outbreak, and elucidate transmission events. In this investigation, the application of the temporal scan statistic identified several significant CDI clusters, two of which were potential outbreaks. Furthermore, significant increases in the incidence rate of CDI cases in years 2007 and 2010 were concordant with the findings from the temporal scan statistic as approximately 67% of the clusters identified spanned 2007 or 2010.

The application of the scan statistic to retrospective microbiology and patient data can help enhance infection control activities in the hospital setting. For example, at the study hospital, specific threshold criteria are currently being used to determine if a *C. difficile* outbreak is occurring and subsequently, if an investigation or response should be initiated. By employing a statistical method for hospital surveillance, threshold criteria may be created or re-defined for detecting *C. difficile* outbreaks that are specific to a particular medical facility, service, or ward. In addition, the application of the scan statistic, prospectively, may result in the identification of a potential cluster or outbreak in a timely manner. Subsequently, infection control activities may be initiated earlier to prevent additional cases. Future studies examining the utility of the temporal scan statistic for identifying CDI clusters under different settings (e.g., hospital type, length of investigation, retrospective and prospective analyses) and comparing results to other surveillance algorithms are required.

## Abbreviations

CDI: *Clostridium difficile* infection; IPC: Infection prevention and control; AIC: Akaike’s information criteria; tcdA: Toxin A; tcdB: Toxin B; cdtA: Binary toxin; IRR: Incidence rate ratio; CI: Confidence interval; n: Number of isolates.

## Competing interests

The authors declare that they have no competing interests.

## Authors’ contributions

MCF contributed to study design, data collection, analysis, and drafting of the manuscript. DLP and OB contributed to study design and statistical analysis. WAC contributed to study design and data collection. JSW contributed to study design and molecular analysis. RRS contributed to study design. All authors contributed to the editing and final version of the manuscript.

## Pre-publication history

The pre-publication history for this paper can be accessed here:

http://www.biomedcentral.com/1471-2334/14/254/prepub
